# Exposure to cosmetic talc and mesothelioma

**DOI:** 10.1186/s12995-023-00367-5

**Published:** 2023-01-18

**Authors:** Jacqueline Moline, Kesha Patel, Arthur L. Frank

**Affiliations:** 1 Department of Occupational Medicine, Epidemiology and Prevention, Northwell Health, Great Neck, NY 11021 USA; 2 Donald and Barbara Zucker School of Medicine at Hofstra/Northwell, Hempstead, NY 11549 USA; 3 Dornsife School of Public Health of Drexel University, Philadelphia, PA 19104 USA

**Keywords:** Malignant mesothelioma, Cumulative asbestos exposure, Cosmetic talcum powder

## Abstract

**Aim:**

Mesothelioma is associated with asbestos exposure. In this case series, we present 166 cases of individuals who had substantial asbestos exposure to cosmetic talc products as well as some who had potential or documented additional exposures to other asbestos-containing products and who subsequently developed mesothelioma.

**Methods:**

Data were gathered for all subjects referred to an occupational and environmental medicine specialist as part of medicolegal review. Years of total cosmetic talcum powder usage was noted as well as the latency from the onset of talcum powder use to the mesothelioma diagnosis. Alternate asbestos exposure in addition to the exposure from cosmetic talc was categorized as none, possible, likely, and definite.

**Results:**

In 122 cases, the only known exposure to asbestos was from cosmetic talc. For 44 cases, potential or documented alternate exposures in addition to the cosmetic talc were described.

**Conclusion:**

Cumulative exposure to asbestos leads to mesothelioma; for individuals with mixed exposures to asbestos, all exposures should be considered. Use of cosmetic talc is often overlooked as a source of asbestos exposure. All individuals with mesothelioma should have a comprehensive history of asbestos exposure, including cosmetic talc exposure.

## Introduction

Mesothelioma, described as a sentinel tumor, is intimately associated with asbestos exposure. Asbestos has been used for decades in thousands of products, both in occupational and non-occupational settings, historically accounting for the bulk of mesothelioma cases. Non-occupational exposures can be environmental in nature, from effluents from mines and factories, from para-occupational exposures such as “shade-tree mechanics” using friction products, and from home renovations [1]. Household exposures affecting family members, known as “take-home” exposure has been well described in the literature [2–5]. An underappreciated source of exposure is the use of cosmetic talc products. The International Agency for Research on Cancer (IARC) [6] states that asbestos contaminated talc is carcinogenic and should be treated as if one were dealing with asbestos. Asbestos levels in talcum powder are significantly above background ambient asbestos exposure levels [7–9]. Talc application simulation studies have been published [7, 8] where exposures to talcum powder were 1.9 f/cc and 2.57 f/cc, respectively. According to the Gramond et al. [10] categorization of intensity, asbestos exposure at these levels would be considered to be high (> 1–10 f/ml).

Historically, asbestos exposures at work have been linked to multiple products. The overall risk for asbestos related disease, including mesothelioma, is related to cumulative exposure. As agencies such as NIOSH, OSHA, the EPA, and others have recognized, there is no known safe exposure to asbestos. Low doses of exposure to asbestos contribute to mesothelioma [11]. Both time from first exposure (latency) and total exposure (cumulative dose) to asbestos must be taken into account when evaluating risk. With multiple repeated incidences of exposure, all those above background level should be thought of as “’substantial.” When considering the elevated risk of mesothelioma in sheet metal workers [12, 13], Zoloth and Michaels considered the multiple bystander exposures to different products, not simply one construction material. This case series presents 166 cases of individuals who had a minimum of five years and a mean of 40.8 years of exposure to asbestos through cosmetic talc products, some with possible other exposures, but all developed mesothelioma.

## Methods

Data were gathered for all subjects referred to an occupational and environmental medicine specialist, JM, as part of medicolegal review. All cases were reviewed personally by an occupational medicine specialist with experience evaluating asbestos exposure in thousands of individuals. The individual’s medical records were reviewed and mesothelioma diagnoses were based on pathological reports that were performed as part of their diagnostic evaluation. Exposure data was obtained by sworn testimony of the mesothelioma patients in all cases, and/or from family members who had direct knowledge of the individual’s use of cosmetic talc and, if present, other sources of asbestos exposure. Use of talc was recorded as being diapered or powdered as a child; diapering or powdering children or others; applying talcum powder to oneself after bathing, or other personal applications of talc. Years of total cosmetic talcum powder usage was noted as well as the latency from the onset of talcum powder use to the mesothelioma diagnosis. Age was presented within a 10 year window to maintain confidentiality. Data reviewed included family occupational histories (parents or anyone cohabitating with the individual), hobbies, residence, living with or laundering clothes of an asbestos exposed worker, if indicated, home renovations that could have exposed the individual to asbestos containing construction materials, residence close to a facility with environmental contamination, or other potential asbestos exposures. In those individuals with potential asbestos exposure in addition to the cosmetic talc, categorization of these exposures was done by two occupational physicians, JM and ALF. Alternate asbestos exposure in addition to the exposure from cosmetic talc was categorized as none, possible, likely, and definite following the descriptions by Gramond et al. [10]. Non-occupational exposure to asbestos was characterized as paraoccupational (living with an asbestos worker or cleaning clothes), do-it-yourself home repair, domestic (handling asbestos material or living in the presence of asbestos material susceptible to damage at home), or environmental (living near and asbestos processing plant). This study was conducted with approval from the Human Research Protection Program at Northwell Health Feinstein Institute for Medical Research (#21–0897-OTH).

## Results

We identified 166 individuals with exposure to cosmetic talc who were diagnosed with a malignant mesothelioma between 2014 and 2021. None of these individuals were previously included in publications by the authors [14]. A summary of the case findings is found in Table 1. Overall, the average age of diagnosis was 63.3 (age range 26–94) years of age. The majority of cases were epithelioid mesothelioma (75.3%). The average length of exposure to cosmetic talc was 40.8 years (range 5–76 years of use), and the average latency period from the onset of talcum powder use to the development of mesothelioma was 52.4 (20—83 years). We identified 122 individuals with asbestos exposure solely through use of cosmetic talc. Exposure to talcum powder could have been for personal use, in an occupational setting (for example, a nurse applying talcum powder to a patient), or applying talcum powder to others such as children. For 122 individuals, they either used cosmetic talc while diapering children or recalled applying talc to others (such as their children). Overall, 80.6% of women and 52.4% of men used talcum powder for diapering or applying talc to others. For 44 individuals, potential alternate asbestos exposure in addition to cosmetic talc was reported. Table 1 presents the 44 cases with alternate exposure ranked by possible, likely, and definite asbestos exposure. Twenty-two women (17.8%) and fifteen men (35.7%) had likely or definite alternate exposure to asbestos in addition to their talcum powder usage. [Details of the exposure history of all 166 individuals with cosmetic talc exposure is presented in Table 2, including a description of the alternate exposure.] Table 1  also shows the site of the tumor by gender. Of the 166 cases, 109 were pleural, 52 were peritoneal, 4 were discovered in both the pleura and peritoneum and the original site could not be determined. One case of pericardial mesothelioma was noted out of the 166 cases, which reflects the rarity of this site for mesothelioma. The percentages of peritoneal mesothelioma were similar for women (29.8%) and men (35.7%). The high proportion of peritoneal mesothelioma tumors relative to pleural tumors, consistent with prior case series of patients with malignant mesothelioma after cosmetic talc use [14, 15], is unusual and deserves further investigation.

**Table 1:** Characteristics of 166 mesothelioma cases with cosmetic talc usage

		**Total (*N* = 166)**	**Female (*n* = 124)**	**Male (*n* = 42)**
**Average Age (range)**		63.3 (26 – 94)	64.3 (26 – 94)	60.9 (28 – 83)
Years of Talc Use^*^ (range)		40.8 (5 – 76)	40.4 (6 – 76)	42.0 (5 – 74)
**Talc Latency in Years (range)**		52.4 (20 – 83)	53.3 (20 – 83)	49.9 (28 – 74)
Diapering or Applying Talc to Others^**^		122 (73.5%)	100 (80.6%)	22 (52.4%)
**Talc Use Only**		122 (73.5%)	97 (78.2%)	25 (59.5%)
**Talc Use and Alternate Exposure**		44 (26.5%)	27 (21.8%)	17 (40.5%)
**Certainty of Alternate Exposure (*n* = 44) (26.5%)**	Possible	7 (4.2%)	5 (4.0%)	2 (4.8%)
	Likely	17 (10.2%)	14 (11.3%)	3 (7.1%)
	Definite	20 (12.0%)	8 (6.5%)	12 (28.6%)
**Tumor Location**	Pleura	109 (65.7%)	83 (66.9%)	26 (61.9%)
	Peritoneum	52 (31.3%)	37 (29.8%)	15 (35.7%)
	Both Pleura & Peritoneum	4 (2.4%)	3 (2.4%)	1 (2.4%)
	Pericardium	1 (0.6%)	1 (0.8%)	0
**Tumor Subtype**	Biphasic	24 (14.5%)	18 (14.5%)	6 (14.3%)
	Epithelial	125 (75.3%)	92 (74.2%)	33 (78.6%)
	Sarcomatoid	16 (9.6%)	13 (10.5%)	3 (7.1%)
	Not specified	1 (0.6%)	1 (0.8%)	0

^*^Years of Talc Use: includes years of being diapered or powdered with talc as a child; years of diapering or powdering children or others with talc; and years applying talcum powder to oneself after bathing or other personal use

^**^Diapering or Applying Talc: restricted to diapering or powdering children with talc or applying talcum powder to others, including occupational use

**Table 2:** Description of 166 mesothelioma cases

Age at Diagnosis	Sex	Tumor Location	Tumor Subtype	Occupation(s)	Talc Latency (years)	Years of Talc Use^*^	Diapering/ Applying Talc to Others^**^	Certainty of Alternate Exposure	Type of Alternate Exposure
51–60	F	Pleura	Epithelial	Cosmetics factory	41	36	Yes	None	
31–40	F	Peritoneal	Biphasic	Marketing	39	12	No	None	
91–100	F	Pleura	Epithelial	Clerical worker	69	57	Yes	Definite	Smoked Kent cigarettes in 1950s
51–60	M	Pleura	Biphasic	Warehouse supervisor	54	22	No	Definite	Home renovations as child
21–30	M	Peritoneal	Epithelial	Aircraft technician	28	5	No	None	
41–50	F	Pleura	Biphasic	Marketing	47	47	Yes	Definite	Automotive friction exposure
41–50	F	Peritoneal	Epithelial	Operator technician	37	36	Yes	Likely	Parents worked in chemical plant/with automotive friction materials; no work clothes laundered at home
61–70	F	Peritoneal, pleura	Epithelial	Hairdresser	65	57	Yes	Definite	Household exposures laundering clothes (automotive friction materials)
41–50	F	Pleura	Epithelial	Industrial engineer	45	10	Yes	None	
71–80	M	Pleura	Biphasic	Firefighter, painter	59	59	No	Definite	Occupational exposures to industrial talc, firefighting
51–60	F	Peritoneal	Epithelial	Dental assistant, secretary, logging business	58	57	Yes	Definite	Automotive friction product exposure
51–60	F	Peritoneal, pleura	Epithelial	Nurse	50	20	Yes	None	
71–80	F	Pleura	Epithelial	Secretary	60	61	No	None	
61–70	M	Peritoneal	Epithelial	Software engineer	53	53	No	Likely	Construction work as teenager; family member machinist
61–70	F	Pleura	Epithelial	Secretary	61	20	Yes	None	
61–70	M	Pleura	Epithelial	Law professor	46	45	No	None	
21–30	F	Peritoneal	Biphasic	Customer service manager	26	12	No	None	
51–60	F	Pleura	Epithelial	Dental assistant, sales	50	17	Yes	Likely	Dental tape used in office
21–30	F	Pleura	Epithelial	Programmer	29	17	Yes	None	
51–60	F	Pleura	Epithelial	Clerical worker	54	48	No	None	
71–80	F	Pleura	Biphasic	Dental assistant, receptionist	64	34	Yes	Possible	Possible household exposure from parental occupations
61–70	F	Pleura	Epithelial	Systems analyst	62	61	Yes	None	
51–60	F	Peritoneal	Epithelial	Clerical worker	59	31	Yes	Likely	Asbestos shingle exposure as child
71–80	F	Peritoneal	Epithelial	Teacher's aide, customer service	46	46	No	None	
51–60	M	Pleura	Epithelial	Baked goods manufacturer	55	49	Yes	None	
61–70	F	Pleura	Epithelial	Housekeeping, packaging	51	23	No	None	
41–50	M	Peritoneal	Epithelial	Lawyer	46	23	Yes	None	
51–60	M	Pleura	Sarcomatoid	IT	30	30	Yes	None	
61–70	F	Peritoneal	Epithelial	Bookkeeper	62	15	Yes	None	
71–80	M	Pleura	Epithelial	Engineer	71	20	No	Definite	Home renovations, automotive friction products, cement in molds
41–50	F	Pleura	Epithelial	Restaurant	46	12	No	None	
81–90	F	Pleura	Epithelial	Not provided	62	62	Yes	Likely	Household exposures laundering clothes (automotive friction materials)
81–90	F	Pleura	Epithelial	LPN	67	18	Yes	Likely	Household exposures laundering clothes (automotive friction materials)
31–40	F	Peritoneal	Epithelial	Nanny, teacher	26	7	Yes	Definite	Home renovations
61–70	M	Peritoneal	Epithelial	Packaging, machine operator, welding	48	48	Yes	Definite	Cut transite and cement pipes; automotive friction exposure (“shade tree”)
71–80	F	Peritoneal	Biphasic	Clerical worker	61	39	Yes	None	
51–60	F	Peritoneal	Epithelial	Lawyer	54	18	No	None	
41–50	F	Pleura	Sarcomatoid	Research	44	45	Yes	None	
51–60	F	Peritoneal	Epithelial	Variety of jobs	50	48	Yes	Definite	Home renovation
41–50	M	Peritoneal	Epithelial	Farrier, mechanic, general labor	41	35	Yes	Definite	Occupational exposure
81–90	M	Pleura	Epithelial	Barber	50	36	Yes	Likely	Boiler work in rail yards
71–80	M	Pleura	Biphasic	Bus driver, factory worker	47	47	Yes	None	
51–60	F	Peritoneal	Epithelial	Physician	20	12	Yes	None	
61–70	F	Pleura	Epithelial	Cashier, sales, clerical worker, wire assembler	53	53	No	None	
51–60	F	Peritoneal	Epithelial	Laborer	41	50	Yes	Likely	Home renovations, family member worked with clay
61–70	M	Pleura	Epithelial	Accountant	69	69	Yes	Definite	Home renovations during 1970s
81–90	F	Pleura	Biphasic	Bookkeeping, rehab counseling	83	32	Yes	None	
71–80	F	Pleura	Sarcomatoid	Office manager	75	75	Yes	None	
61–70	F	Pleura	Epithelial	Merchandising manager	31	18	Yes	None	
41–50	F	Pleura, peritoneal	Epithelial	Teacher	46	22	Yes	None	
51–60	M	Pleura	Epithelial	Automechanic, pipefitter	31	44	Yes	Definite	Occupational and take home exposure (shipyard)
51–60	F	Peritoneal	Epithelial	Clerical worker	40	38	Yes	None	
41–50	F	Pericardium	Sarcomatoid	Medical center	50	31	Yes	None	
71–80	M	Pleura	Epithelial	Mechanic, parts manager	61	50	No	Definite	Occupational naval exposure to asbestos, automotive friction material handling
71–80	F	Pleura	Epithelial	Secretary, cosmetics, cashier	50	25	No	Definite	Household exposures laundering clothes (automotive friction materials)
61–70	F	Pleura	Sarcomatoid	Catering	45	40	Yes	None	
61–70	F	Pleura	Epithelial	Cleaner, personal assistant	52	50	Yes	None	
51–60	M	Pleura	Epithelial	Meat inspector	41	26	No	None	
71–80	F	Pleura	Epithelial	Office manager	65	55	Yes	Possible	Household exposure from husband (drilling wells, pipes)
71–80	F	Pleura	Epithelial	Clerical worker	60	59	Yes	Possible	Family member worked at service station (no details on work)
71–80	M	Pleura	Sarcomatoid	Accountant	47	39	Yes	None	
61–70	F	Pleura	Epithelial	Sales, business	60	16	No	None	
81–90	M	Peritoneal	Epithelial	Accountant	68	56	No	None	
51–60	F	Pleura	Epithelial	Social worker	40	6	No	None	
71–80	F	Pleura	Epithelial	Hairdresser	60	49	Yes	None	
41–50	M	Pleura, peritoneal	Epithelial	Warehouse worker	47	8	No	Definite	Automotive filler exposure
51–60	F	Pleura	Epithelial	Retail, bankteller, work at school	56	56	Yes	None	
61–70	F	Pleura	Biphasic	Bakery	64	49	Yes	None	
71–80	F	Pleura	Epithelial	Hospitality	61	58	Yes	None	
51–60	F	Pleura	Epithelial	Cashier	57	56	Yes	None	
81–90	F	Peritoneal	Epithelial	Teacher	40	50	Yes	Possible	Abatement done at work
81–90	F	Pleura	Epithelial	Physical therapy assistant	64	62	Yes	None	
31–40	M	Peritoneal	Epithelial	IT	35	25	No	None	
51–60	F	Pleura	Epithelial	Housekeeper	42	34	Yes	None	
81–90	F	Pleura	Epithelial	Teacher	54	50	Yes	Likely	Home renovations
61–70	M	Pleura	Epithelial	Accounting	63	63	Yes	None	
71–80	M	Pleura	Epithelial	Tractor driver, race track	62	60	No	Likely	Oil drilling
71–80	F	Pleura	Epithelial	Research	62	42	Yes	None	
51–60	F	Peritoneal	Epithelial	Agriculture consultant	53	53	Yes	None	
31–40	F	Peritoneal	Epithelial	Clerical worker	39	27	Yes	None	
41–50	F	Pleura	Biphasic	Clerical worker	49	49	No	Likely	Ceramics use
71–80	M	Pleura	Epithelial	Communications system/office	47	49	Yes	None	
81–90	F	Pleura	Not specified	Nurse	76	70	Yes	Likely	Family member worked in shipyard
71–80	F	Peritoneal	Epithelial	Meat wrapper	56	47	Yes	None	
71–80	M	Pleura	Biphasic	Advertising	62	62	Yes	None	
71–80	F	Peritoneal	Epithelial	Teacher, hospital administration	70	70	Yes	None	
41–50	M	Peritoneal	Epithelial	Chef	37	36	No	None	
51–60	F	Peritoneal	Epithelial	Case manager	53	52	Yes	None	
41–50	F	Peritoneal	Epithelial	Finance and marketing	42	34	No	None	
51–60	M	Pleura	Epithelial	Painter, carpet installer	38	38	Yes	Definite	Automotive friction product use
81–90	F	Pleura	Epithelial	Secretary	63	45	Yes	None	
71–80	F	Pleura	Epithelial	Nursing	69	40	Yes	None	
51–60	F	Peritoneal	Epithelial	Nurse	46	22	Yes	None	
41–50	M	Peritoneal	Epithelial	Industrial engineer	48	53	Yes	None	
71–80	F	Peritoneal	Epithelial	Librarian	55	44	Yes	None	
51–60	F	Pleura	Biphasic	Clerical worker, hostess	53	15	No	None	
71–80	F	Pleura	Epithelial	Secretary	67	55	Yes	None	
71–80	F	Pleura	Epithelial	Clerical worker	64	20	Yes	None	
71–80	F	Peritoneal	Sarcomatoid	Secretary, medical billing	47	47	Yes	None	
81–90	F	Pleura	Epithelial	Communications and real estate	69	69	Yes	None	
41–50	F	Peritoneal	Epithelial	Home health	42	24	Yes	None	
51–60	F	Pleura	Biphasic	Clerical worker, hostess	53	15	Yes	None	
71–80	F	Pleura	Biphasic	Therapy aid	67	67	Yes	None	
61–70	M	Pleura	Biphasic	Restaurant, lead technician	50	47	Yes	None	
61–70	F	Pleura	Sarcomatoid	Teacher	51	43	Yes	None	
51–60	F	Pleura	Epithelial	Sales	44	16	Yes	None	
41–50	F	Pleura	Epithelial	Chicken farming, medical assistant	48	48	Yes	None	
41–50	M	Peritoneal	Epithelial	Physician	40	17	No	None	
71–80	F	Pleura	Epithelial	Secretary	69	64	No	None	
61–70	F	Pleura	Epithelial	Assembly line worker	51	52	Yes	None	
61–70	F	Pleura	Epithelial	X-ray technician	40	40	Yes	None	
51–60	F	Pleura	Biphasic	Factory worker, housekeeper	33	14	Yes	None	
51–60	F	Pleura	Epithelial	Clerical worker	38	29	Yes	None	
61–70	F	Pleura	Epithelial	Clerical worker	54	27	Yes	None	
81–90	F	Pleura	Epithelial	Variety of jobs	76	76	Yes	None	
71–80	F	Peritoneal	Epithelial	Receptionist, dental assistant	45	35	Yes	None	
71–80	F	Pleura	Epithelial	Midwife	55	47	Yes	None	
81–90	F	Pleura	Epithelial	Seamstress	63	56	Yes	None	
71–80	F	Peritoneal	Epithelial	Accounting	65	65	Yes	None	
61–70	F	Pleura	Epithelial	Nurse	57	57	Yes	None	
71–80	M	Peritoneal	Epithelial	Sales, truck driver	45	32	No	Definite	Automotive friction products use
51–60	M	Pleura	Epithelial	Lawyer	48	48	Yes	None	
51–60	F	Pleura	Sarcomatoid	Customer service	56	27	Yes	None	
81–90	F	Pleura	Sarcomatoid	Teacher	53	47	Yes	None	
71–80	F	Peritoneal	Epithelial	Not provided	67	48	No	Definite	Smoked Kent cigarettes in 1950s
31–40	F	Peritoneal	Biphasic	Banking	39	19	Yes	None	
71–80	M	Pleura	Epithelial	Logger, run loader	74	74	Yes	Possible	Automotive friction product use and home renovations
71–80	F	Peritoneal	Epithelial	Hairdresser	50	50	Yes	Likely	Hairdryers present in salon
31–40	F	Pleura	Sarcomatoid	Certified Nursing Assistant and phlebotomist	39	24	Yes	None	
71–80	F	Pleura	Epithelial	Seamstress	62	52	Yes	None	
31–40	F	Peritoneal	Epithelial	Variety of jobs	27	20	Yes	None	
61–70	F	Pleura	Epithelial	Laborer	51	51	Yes	None	
41–50	M	Peritoneal	Biphasic	Casino worker	47	47	Yes	None	
81–90	F	Pleura	Epithelial	Nurse	67	18	Yes	None	
71–80	M	Pleura	Epithelial	Accountant, comptroller	70	30	Yes	Possible	Home renovations
61–70	F	Pleura	Sarcomatoid	Clerical worker	60	27	No	None	
61–70	F	Pleura	Biphasic	Secretary, cleaner	52	52	Yes	Likely	Home renovations in 1970s
61–70	F	Pleura	Sarcomatoid	Office cleaner, food prep	64	64	Yes	None	
61–70	F	Pleura	Epithelial	Insurance agent	58	57	Yes	None	
71–80	F	Pleura	Epithelial	Cook, cleaner, concierge	54	44	Yes	None	
31–40	M	Peritoneal	Epithelial	Lab technician	36	36	No	None	
91–100	F	Pleura	Epithelial	Variety of jobs	60	60	No	None	
51–60	F	Pleura	Epithelial	Real estate broker	36	35	Yes	None	
41–50	F	Pleura	Biphasic	PhD in astronomy, dance teacher	37	37	No	None	
71–80	F	Pleura	Epithelial	Switchboard operator, HR	64	50	No	None	
51–60	F	Peritoneal	Epithelial	Nurse	50	46	Yes	Likely	Ceramics work for 4–5 years
41–50	F	Peritoneal	Epithelial	Lawyer	44	20	No	None	
71–80	F	Pleura	Epithelial	Quality control	67	67	Yes	None	
51–60	F	Pleura	Sarcomatoid	Counselor	55	54	No	None	
71–80	F	Pleura	Biphasic	Certified nursing assistant, ranch work	62	35	Yes	Likely	Vermiculite exposure
71–80	M	Peritoneal	Epithelial	Physician	67	67	Yes	None	
41–50	F	Peritoneal	Epithelial	Nurse Practitioner	44	34	Yes	None	
31–40	M	Pleura	Epithelial	Not provided	29	30	No	None	
61–70	F	Pleura	Epithelial	Banking	60	50	Yes	None	
51–60	F	Peritoneal	Epithelial	Nurse	56	45	Yes	None	
71–80	F	Pleura	Sarcomatoid	Hairdresser	61	25	Yes	None	
71–80	M	Pleura	Sarcomatoid	Mechanic	60	48	No	Definite	Occupational exposure and home renovations
61–70	M	Peritoneal	Epithelial	Worked at special education preschool	57	50	No	None	
71–80	F	Peritoneal	Biphasic	Teacher	65	46	Yes	None	
71–80	F	Pleura	Biphasic	Teacher	71	59	Yes	Possible	Family member was Linotype operator
81–90	F	Pleura	Epithelial	Cashier, waitress	60	58	Yes	None	
31–40	F	Pleura	Epithelial	Administrator	28	7	No	None	
71–80	F	Peritoneal	Epithelial	Teacher	50	51	Yes	Likely	Household exposure to laundry (automotive friction materials)
31–40	M	Pleura	Epithelial	Homeland Security	33	33	No	None	
61–70	M	Pleura	Epithelial	Trucking company	55	56	Yes	None	
71–80	F	Pleura	Epithelial	Office worker	62	45	Yes	None	

^*^Years of Talc Use: includes years of being diapered or powdered with talc as a child; years of diapering or powdering children or others with talc; and years applying talcum powder to oneself after bathing or other personal use

^**^Diapering or Applying Talc: restricted to diapering or powdering children with talc or applying talcum powder to others, including occupational use

## Discussion

This paper presents 166 individuals with malignant mesothelioma and asbestos exposure through documented use of cosmetic talcum powder. For 122 of 166, their only known exposure to asbestos was their use of cosmetic talcum powder. Without the recognition of asbestos exposure through cosmetic talcum powder, 73.5% of the cases might well have been considered “idiopathic.” Similarly, for those 26.5% of cases with additional asbestos exposure along with the talc, those alternate exposures would have been mistakenly considered as the sole, and sufficient, cause of the mesothelioma. Historically, the attributable risk of asbestos for mesothelioma in women ranged from around 20–50%. However as Baur et al. point out, misclassification or inadequate exposure ascertainment has led to this low attributable risk for women compared to men. [16]. Data from occupationally exposed cohorts that included men and women actually show that compared to similarly exposed men, women had higher mortality rates from mesothelioma [17–20]. Lacourt found that at low-level cumulative asbestos exposure ((0 – 0.1 f-cc/year) women were more likely to develop mesothelioma than men [21]. Magnani (2008) found the SMR for mesothelioma was higher for women than for men among workers at an asbestos cement plant [22]. Frank et al. (2009) found mesothelioma rates in the Qingdao region of China were correlated with a higher proportion of women employed in asbestos manufacturing industries. [23] In some instances authors limited the characterization of asbestos exposure in women to certain industries, such as shipbuilding during wartime [24], thus neglecting other potential sources and decreasing the attributable risk. Conversely, when non-occupational exposures were included for women, even with low-intensity domestic exposure considered, the attributable risk increased from 40% to 64.8% [21].

Given that all types of asbestos can cause mesothelioma [6], it is important to consider every source of exposure to asbestos in an individual. Talcum powder has been contaminated with both chrysotile and amphibole asbestos (predominately anthophyllite and tremolite) [8, 25, 26]. Recently, Wong et al. (2021) found significantly elevated risks of mesothelioma among individuals with only chrysotile exposure and for mixed fiber exposure. [27]. Chrysotile alone (OR = 3.8) and in combination with tremolite/anthophyllite asbestos (OR = 3.9) were associated with similar increases in risk of mesothelioma. These three fiber types are most commonly found in cosmetic talc, and given that different ore sources were used in manufacturing over time, it is likely that many formulations and uses of talcum powder involved mixed fiber type exposure. There is no scientific basis to state that one type of exposure was the sole cause of the mesothelioma in a mixed exposure scenario. For example, rates of mesothelioma have been evaluated based on either job type or locale (e.g., construction, shipping) rather than on each specific task or exposure within the job category. Furthermore, mesothelioma is a disease that occurs following a long latency period. It is important to consider whether the latency period for all exposures, whether due to asbestos in talcum powder, or through occupational or para-occupational exposures meets the minimum latency period.

Subgroups of individuals not traditionally known to be exposed to asbestos have been identified, such as teachers. In this case series, 12 teachers (7.2% of cases) were diagnosed with mesothelioma. Anderson et al. identified 12 school teachers with mesothelioma in Wisconsin (6 male, 6 female). [28]. Nine cases had no known exposure to asbestos, although several worked in school buildings with asbestos containing building materials (ACBM) present, but the condition of the ACBR while the teachers were present in the school was unknown. No history of talcum powder use was elicited. Marianaccio et al. identified mesotheliomas in 11 female teachers in Italy. [29]. Mazurek et al. evaluated mesothelioma deaths in women in the United States from 1999–2020 using death certificate data. [30]. Mesothelioma was noted in 32 female elementary and middle school teachers. No information on exposure to asbestos or specific tasks at work or a comprehensive exposure history was available; no history of talcum powder use was elicited, as the study was based solely on death certificates. Tomasallo et al. found increased mortality among school teachers in Wisconsin, USA. [31]. They noted that para-occupational or take home exposure could be responsible for the increased risk. Again, no history of asbestos exposure through talcum powder usage was ascertained. It might be possible that exposure to ACBM played some role in these mesotheliomas, however, the notable history of exposure to asbestos-containing talcum powders among teachers in this case series, highlights the importance of assessing this source of exposure in future studies of mesothelioma in teachers and other predominantly female professions.

Mazurek et al. found seven cases of mesothelioma among female hairdressers. [30]. Our series identified five hairdressers/barbers with documented occupational exposure to asbestos containing talcum powder. Moline et al. found three hairdressers who used cosmetic talc as part of their occupation, [14] and Emory et al. [15] found 4 hairdressers out of 75 patients. Pavlisko et al. identified a hairdresser in their study of mesothelioma in women, but classified the case in the non-occupational/paraoccupational exposure category. [32] McDonald attributed the finding of tremolite in the lung tissue of a chrysotile worker to his prior occupational exposure to talc as a barber [33]. Rodelsberger recognized talc as a source of asbestos exposure and identified hairdressers and barbers as asbestos-exposed industries [34]. The examples of these two occupational subgroups, teachers with personal use of cosmetic talc, and hairdressers with occupational use of cosmetic talc, show the importance of obtaining a thorough history and determining all potential sources of asbestos exposure.

This case series describes mesotheliomas in end-users of cosmetic talcum powder, thus using no personal protective equipment or dust suppression activities, unlike some cohorts with occupational exposures [35]. Prior mortality studies of talc miners and millers in Italy (and other countries) have not identified mesotheliomas in their populations, although two cases of peritoneal cancer were identified by Pira et al. [36]. The Rubino, Coggiola and Pira et al. studies used mortality data collected prior to an ICD mesothelioma code, which could impact proper classification of mesothelioma. [35–37]. The studies had a relatively small sample size, which given the rarity of mesothelioma, even among highly exposed individuals, would have led to insufficient statistical power [38]. Fordyce studied Vermont talc miners and found two mesotheliomas in the small cohort of 427 miners; Vermont talc has been used in cosmetic talcum powder [39].

Fiber burden studies were done in some individuals from the two prior case series of mesothelioma among individuals with cosmetic talcum powder use. Moline et al. reported on tissue fiber analysis in six of 33 individuals. Asbestos fibers, of the types found in cosmetic talc, were found in all six samples. Emory et al. found anthophyllite asbestos in all 9 individuals for whom tissue fiber analysis was done. Tremolite was found in six of the cases in addition to the anthophyllite. Hull et al. [40] looked at New York State talc miners and found anthophyllite, tremolite/actinolite, chrysotile and talc in their lungs. There were over a dozen cases of mesothelioma identified in these talc miners. Our case series did not include data on tissue sampling, which is not typically done for clinical purposes; rather we relied on patient history. For occupational exposures to asbestos, fiber analysis is not required to ascertain a history of exposure, rather the history of exposure to asbestos is sufficient [41]. This should be no different for environmental exposures, such as asbestos exposure in cosmetic talcum powder, or even para-occupational exposures.

Pleural mesothelioma is more common than peritoneal mesothelioma [42], with estimates of pleural mesothelioma occurring approximately 80–90% of the time compared to peritoneal mesothelioma. The presenting location for the tumor, either pleural or peritoneal, was similar in all three recent case series. In Moline et al., 11 of 33 patients had peritoneal mesothelioma and in Emory et al., 23 of 75 cases were peritoneal mesothelioma. In this larger case series, the proportion of peritoneal mesotheliomas was 31.3%. The proportion of men in each of the three case series was similar. In Emory et al., 15% of the cases were men, compared with 18% of the cases in Moline et al. In the current case series, among 122 cases with talc-only exposure, 20.5% were men, slightly above the proportion in two previous case series. This might reflect growing awareness among men that talcum powder use could explain their mesothelioma, particularly when no other identifiable source of asbestos was identified. Few individuals in this case series underwent testing for the tumor suppressor gene, BAP-1, which is associated with an increased risk for mesothelioma when associated with asbestos exposure, [43] including greater susceptibility at low doses of asbestos such as exposures from cosmetic talcum powder use. Interestingly, there was a greater frequency of peritoneal mesothelioma cases in those with the BAP-1 mutation and asbestos exposure [44].

Several authors have written about the importance of the cumulative dose, which has been related to several asbestos-caused diseases, both non-malignant and non-malignant. Luberto et al. discussed the “increased mortality risk due to asbestos exposure for malignant neoplasm of pleura, peritoneum, lung and ovary, as well as asbestosis, all increasing with cumulative exposure.” [19] Henderson et al. commented on the use of the cumulative exposure model in the Helsinki Criteria. [45] Iwastsubo and colleagues, citing only low exposures leading to disease noted that “excess of mesothelioma was observed for levels of cumulative exposure.” [46] Ferrante and her colleagues [47] found that the “risk of pleural malignant mesothelioma increased with cumulative asbestos exposure and also in analyses limited to subjects non-occupationally exposed,” comparable to the current case series. Albin et al. [48] even noted that “colorectal cancer displayed a clear relation with cumulative dose,” as one would reasonably expect with asbestos-related diseases.

This case series may reflect the potential sources of bias that impact all studies that use cases in which litigation is occurring. However, because mesothelioma is a rare disease and full environmental histories are rarely obtained or documented, it would be impossible to amass so many cases with one type of exposure using standard sources such as hospital or cancer registry records. Furthermore, most patients (and their clinicians) are unaware of the presence of asbestos in talcum powder, leading them to report no known asbestos exposure. The data related to years of exposure to cosmetic talcum powder was obtained and typically described in great detail during sworn testimony. For nearly one-quarter of the individuals in this series, additional exposures to asbestos were reported along with the cosmetic talcum powder. When available, information regarding talcum powder usage was corroborated by sworn testimony of family members. Typically, the questioning of individuals about alternate exposures to asbestos as part of litigation is fairly comprehensive, but it is possible that there were additional, unknown sources. This presents a challenge for any study of asbestos exposure and, in particular, mesothelioma, given the long latency period from the onset of exposure to the development of disease.

## Conclusion

For individuals with exposure to asbestos through cosmetic talc usage and additional alternate sources, all exposures contribute to the development of mesothelioma. Published case reports and case series have identified over 100 individuals whose sole exposure to asbestos was through cosmetic talcum powder usage [14, 15, 49]. Thus, is it critical to obtain a history of all potential exposures to asbestos. In this case series, 122 cases would have had no source of asbestos identified if a history of asbestos-containing cosmetic talc had not been elicited. The other 44 would have likely been misclassified as having only alternate exposures. It is indisputable that asbestos causes mesothelioma, therefore, it is critical to elicit all potential sources of asbestos exposure so that we can better understand, and prevent, future cases of this deadly cancer.

## Data Availability

The datasets generated and/or analyzed during the current study are not publicly available. Individuals of details of cases will not be provided to protect the confidentiality of the cases presented in the study. Efforts to minimized identification, such as describing age in a range were employed.
